# Expert consensus on the combined screening of genes and biomarkers for neonatal diseases

**DOI:** 10.1007/s12519-025-00996-2

**Published:** 2025-12-26

**Authors:** Xin-Wen Huang, Ting Zhang, Zhen-Zhen Hu, Zhi-Guo Wang, Xiao-Ping Luo, Yan-Ling Yang, Lian-Shu Han, Xue-Fan Gu, Guang-Ren Xiao, Bao-Sheng Zhu, Ru-Lai Yang, Wei-Peng Wang, Yong-Lan Huang, Jian-Hui Jiang, Hua Wang, Guo-Li Tian, Qiao-Ling Sun, Xin-Mei Mao, Bin Yu, Wen-Bin Zhu, Pi-Liang Chen, Hai-Li Hu, Hui-Ming Yan, Jing Liu, Wen-Ying Nie, Feng Wang, Ren Cai, Tao Jiang, Xiao-Hua Wang, Fa-Liang Xu, Yu-Lin Zhou, Jian-Ping Yang, Lin Zou, Wei Wen, Yuan-Yuan Kong, Ming-Cai Ou, Ya-Guo Zhang, Yan-Qin Ying, Rong Qiang, De-Hua Zhao, Chen-Lu Jia, Zhi-Xin Zhang, Ben-Qing Wu, Hui Zou, Zheng-Yan Zhao

**Affiliations:** 1 Department of Genetics and Metabolism, Children’s Hospital, National Clinical Research Center for Child Health, Zhejiang University School of Medicine, 3333 Binsheng Road, Binjiang District, Hangzhou 310052, China; 2 National Center for Clinical Laboratories, Beijing Engineering Research Center of Laboratory Medicine, Beijing Hospital, National Center of Gerontology, Institute of Geriatric Medicine, Chinese Academy of Medical Sciences, Beijing 100730, China; 3 Department of Pediatrics, Tongji Hospital, Tongji Medical College, Huazhong University of Science and Technology, Wuhan 430030, China; 4 Department of Pediatrics, Peking University First Hospital, Beijing 100034, China; 5 Department of Pediatric Endocrinology/Genetics, Shanghai Institute for Pediatric Research, Xinhua Hospital, School of Medicine, Shanghai Jiao Tong University, Shanghai 200092, China; 6 Department of Medical Research, Taipei Veterans General Hospital, Taipei 11217, China; 7 Department of Medical Genetics, the First People’s Hospital of Yunnan Province, Kunming 650032, China; 8 Department of Pediatrics, Yunnan Provincial Key Laboratory for Birth Defects and Genetic Diseases, the First People’s Hospital of Yunnan Province, Kunming 650032, China; 9 Department of Health Care, Maternal, Child Health Hospital of Hubei Province, Wuhan 430070, China; 10 Department of Genetics and Endocrinology, Guangzhou Women and Children’s Medical Center, Guangzhou 510623, China; 11 Children Inherit Metabolism and Endocrine Department, Guangdong Women and Children Hospital, Guangzhou 510000, China; 12 Department of Medical Genetics, Hunan Children’s Hospital, Changsha 410007, China; 13 Neonatal Screening Center, Shanghai Children’s Hospital, Shanghai Jiao Tong University, Shanghai 200040, China; 14 Anhui Provincial Maternal and Child Health Care Association, Hefei 230061, China; 15 Neonatal Disease Screening Center, Peking University First Hospital Ningxia Women and Children’s Hospital (Ningxia Hui Autonomous Region Maternal and Child Health Hospital), Yinchuan 750001, China; 16 Department of Medical Genetics, Changzhou Maternity and Child Health Care Hospital, Changzhou 213003, China; 17 Center of Neonatal Screening, Fujian Provincial Maternity and Children’s Hospital, Affiliated Hospital of Fujian Medical University, Fuzhou 350001, China; 18 Neonatal Disease Screening Center, Gansu Provincial Maternity and Child-Care Hospital, Lanzhou 730050, China; 19 Center of Neonatal Disease Screening, Hefei Maternal and Child Health Care Hospital, Hefei 230000, China; 20 Newborn Screening Center of Hunan Province, Hunan Provincial Maternal and Child Health Care Hospital, Changsha 410078, China; 21 Hunan Provincial Key Laboratory of Regional Hereditary Birth Defects Prevention and Control, Changsha Hospital for Maternal & Child Health Care Affiliated to Hunan Normal University, Changsha, China; 22 Neonatal Disease Screening Center, Jinan Maternal and Child Health Care Hospital Affiliated With Shandong First Medical University, No. 2, Jingsan Road, Jianguo Xiao, Shizhong District, Jinan 250000, China; 23 Department of Medical Genetics, Jiangxi Maternal and Child Health Hospital, Nanchang 330006, China; 24 Jiangxi Provincial Key Laboratory of Birth Defect for Prevention and Control, Jiangxi Maternal and Child Health Hospital, Nanchang 330006, China; 25 Department of Medical Genetics, Liuzhou Maternal and Child Health Care Hospital, Liuzhou 545001, China; 26 Genetic Medicine Center, Nanjing Maternity and Child Health Care Hospital, Women’s Hospital of Nanjing Medical University, Nanjing 210004, China; 27 Department of Genetic and Eugenics, Inner Mongolia Autonomous Region Maternal and Child Health Hospital, Hohhot 010020, China; 28 Neonatal Disease Screening Center, Qinghai Provincial Maternal and Child Health Hospital, Xining 810005, China; 29 United Diagnostic and Research Center for Clinical Genetics, Women and Children’s Hospital, School of Medicine and School of Public Health, Xiamen University, Xiamen 361003, China; 30 Department of Child Health Care, Shanxi Children’s Hospital, Taiyuan 030013, China; 31 Department of Laboratory Medicine, The Nineth Hospital Affiliated to Shanghai Jiaotong University School of Medicine, Shanghai 200011, China; 32 Department of Neonatology, Shenzhen Maternity and Child Healthcare Hospital, Shenzhen 518000, China; 33 Department of Newborn Screening Center, Beijing Maternal and Child Health Care Hospital, Beijing Obstetrics and Gynecology Hospital, Capital Medical University, Beijing 100026, China; 34 Neonatal Disease Screening Center, Sichuan Provincial Maternity and Child Health Care Hospital, Chengdu 610000, China; 35 Medical Heredity Research Center, Northwest Women’s and Children’s Hospital, Xi’an 710061, China; 36 Department of Henan Newborn Screening Center, the Third Affiliated Hospital of Zhengzhou University, Zhengzhou 450052, China; 37 Department of Pediatrics, China-Japan Friendship Hospital, Beijing 100029, China; 38 Department of Neonatology, Children’s Medical Center, University of Chinese Academy of Science Shenzhen Hospital, Shenzhen, China

**Keywords:** Combined screening, Disease biomarker, Genetic screening, Newborn screening

## Abstract

**Background:**

Newborn screening (NBS) through disease biomarkers has significantly reduced severe outcomes of congenital disorders. Moreover, exploratory newborn genetic screening programs are increasingly being implemented. This consensus, developed by multidisciplinary experts, aims to standardize the combined screening of genes and biomarkers for neonatal diseases in China, balancing ethical, technical, and clinical considerations.

**Data sources:**

This consensus synthesizes evidence from peer-reviewed literature (PubMed, CNKI, etc.) up to 2024 and integrates clinical experiences from multidisciplinary experts in neonatology, genetics, and laboratory medicine, focusing on disease biomarker-based NBS, newborn genetic screening, and the clinical utility of combined screening.

**Results:**

The consensus defines principles for combined screening: (1) disease/gene selection: 154 disease-causing genes covering 67 inherited metabolic disorders (e.g., amino acid metabolism disorders, organic acid metabolism disorders), prioritized by treatability, onset age (< 5 years), and cost-effectiveness; (2) methodology: integrating dried blood spot biomarker analysis with next-generation sequencing-based targeted capture (coverage > 300 ×), validated by MLPA/Sanger and long-range sequencing for complex variants (e.g., *CYP21A2*, *SLC25A13*); and (3) operational workflow: standardized workflows for informed consent, sample collection/delivery, and result interpretation, with dual reporting of marker and genetic findings within 15 days. Positive cases require family verification and/or other genetic sequencing techniques.

**Conclusions:**

This consensus establishes a practical framework for integrating marker and genetic screening, aiming to improve diagnostic accuracy and achieve rapid and effective interventions, thereby saving lives and reducing the occurrence of severe complications. Implementation requires interdisciplinary collaboration and ongoing quality control to maximize clinical utility.

**Graphic abstract:**

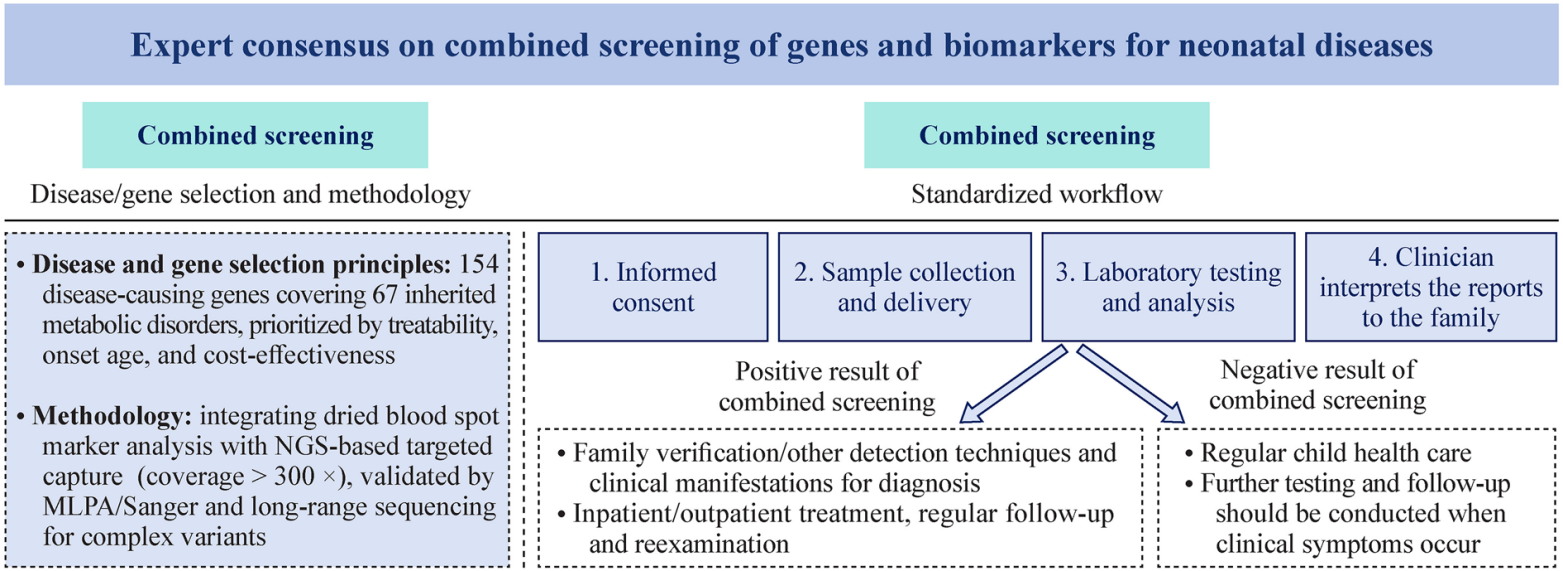

## Introduction

Newborn screening (NBS) through biomarkers enables early diagnosis and treatment, effectively preventing severe disease outcomes. This study also provides guidance for family reproductive planning, reducing the incidence of birth defects and improving population health. This measure has been recognized internationally as one of the "top ten public health achievements" in the first decade of the twenty-first century [1]. Owing to high entry barriers, many low- and middle-income countries (such as Indonesia, Laos, and Albania) still struggle to provide some, if not all, universal NBS [2]. In China, the coverage rate of NBS has exceeded 98%, with biomarker-based screening remaining the dominant method due to its targeted advantage [3]. Moreover, exploratory newborn genetic screening programs are increasingly being implemented [4–8]. To maximize the protection of the rights and interests of screened newborns and their families, the NBS Working Group, in collaboration with multidisciplinary experts from relevant fields of several societies, has jointly formulated the "Expert consensus on combined screening of genes and biomarkers for neonatal diseases". This consensus adheres to the principles of ethical priority, scientific rigor, forward-thinking, and practicality, aiming to provide actionable guidelines for the standardized application and management of combined biomarkers and genetic screening in disease detection.

## Advantages and challenges of disease biomarker-based newborn screening

The international NBS has a history of more than 60 years. With advancements in technologies, such as enzyme-linked immunosorbent assays, time-resolved fluorescence analysis, and tandem mass spectrometry (MS/MS), the range of screened conditions has expanded, and screening efficiency has significantly improved [9, 10]. As a robust approach, traditional disease biomarker-based NBS offers notable strengths: it is cost effective for large-scale population screening, features fast detection speed to meet timely screening needs, and supports high throughput to accommodate the volume of newborns in public health programs. Given that NBS serves as a core public health project, cost effectiveness and operational efficiency are crucial factors that must be prioritized in its nationwide implementation. However, NBS typically relies on dried blood spot (DBS) samples collected on filter paper. Although DBSs can be stored and transported at room temperature, high-temperature or humid conditions may lead to a reduction in or inactivation of enzyme activities [such as glucose-6-phosphate dehydrogenase (G6PD) enzymes] and the degradation of biomarkers (such as Met, Arg, and C2) in filter paper DBSs. Moreover, the accuracy of screening results can be affected by blood spot quality (size, double-spotting, contamination, incomplete saturation, etc.), punch location (center vs. edge of the spot), and hematocrit level [11–14]. Moreover, biomarkers are susceptible to interference from multiple factors, including geographic/ethnic variations [15, 16], seasonal fluctuations [17], neonatal diseases (prematurity, immature development of liver enzymes, liver diseases, immature development of kidneys, jaundice, infections, hypoglycemia, etc.) [18–22], nutrition, therapeutic interventions (extracorporeal life support, blood transfusion, parenteral nutrition, antibiotics, antiepileptic drugs, etc.) [19, 23–25], maternal factors [vitamin B12/carnitine deficiency, maternal inherited metabolic disorders (IMDs)/liver disease, steroid use] [26–28], environmental influences [29], technical limitations of detection methods (MS/MS cannot distinguish isomers, fluorescence assays are affected by anticoagulants, etc.) [30, 31], and cutoff value settings. These variables generate many false negative/false positive results, compromising screening efficiency [32]. More importantly, discrepancies between the results of different biochemical tests have often been observed, enzymatic assays are time-consuming and labor-intensive, and their results are typically semi-quantitative, which further complicates the interpretation of findings [33, 34]. For screen-positive cases, further confirmation requires specialized biochemical testing and genetic testing, leading to a prolonged diagnostic process. This delay may, to some extent, miss the optimal window for early intervention, resulting in irreversible harm to infants.

## Advantages and challenges of newborn genetic screening

Newborn genetic screening is being increasingly implemented worldwide, primarily through three models: (1) two-tier newborn genetic screening: for samples that test positive in initial biomarker screening, direct genetic diagnostic techniques such as quantitative polymerase chain reaction (qPCR) or MassArray nucleic acid mass spectrometry are employed, effectively reducing false-positive rates in primary screening [35–37]; (2) disease-specific newborn genetic screening: NBS for conditions such as deafness [38], sickle cell anemia, and spinal muscular atrophy [39, 40] enables early diagnosis of diseases that cannot be detected through biomarker screening; and (3) next generation sequencing (NGS)-based newborn genetic screening: in September 2013, the United States launched the Newborn Sequencing Initiative, utilizing whole-exome sequencing (WES) and whole-genome sequencing (WGS), followed by four major projects. Multiple studies in other countries and institutions in China have also investigated newborn genetic screening programs one after another [8, 41–49]. The First International Conference on Neonatal Sequencing (ICoNS, 2022, Boston, USA) and the International Conference on Newborn Genomic Screening (2023, London, UK) presented various national newborn genetic screening initiatives from the United States, Europe, and Australia, significantly advancing the application of genetic sequencing in NBS.

On the basis of the current research status of newborn genetic screening, WES, WGS, and targeted gene panels demonstrate relatively high diagnostic yields and clinical utility for critically ill neonates in neonatal intensive care units (NICUs) and hospitalized newborns. In particular, rapid WGS or WES, which reduces sequencing and interpretation time, has proven to be an effective diagnostic tool for critically ill newborns and infants suspected of having a genetic disorder, with reported diagnostic yields ranging from 30% to 57% [50]. However, owing to certain limitations in genetic sequencing technologies (e.g., detection of structural variants and pseudogenes) and variant interpretation [e.g., interpretation of variants of uncertain significance (VUS)], the sensitivity and specificity of screening may be compromised, resulting in a certain degree of false-negative and false-positive findings. In addition, there remains controversy regarding how to report carrier status identified through genetic screening. Consequently, newborn genetic screening cannot fully replace traditional NBS and should serve only as a complementary approach. Furthermore, owing to variations in research objectives, target populations (including NICU patients and hospitalized newborns) [51, 52], diverse technologies, methods and solutions selected by combined NBS (such as WES, WGS, gene panels, hotspot mutation detection, and qPCR), and highly heterogeneous disease selection criteria [53, 54], a standardized system for newborn genetic screening has yet to be established.

## Advantages of combining newborn disease biomarkers with genetic screening

The integration of genetic sequencing into NBS is increasingly recognized as a valuable addition, with pilot programs underway in some regions. However, large-scale implementation depends on local economic conditions and healthcare capacity, making the key questions “where, when, and how” to implement it effectively. In NBS, screening for more diseases is not necessarily better, as expanding the scope of screening introduces greater complexity in terms of technical, psychological, social, economic, and ethical considerations. Only when implemented in conjunction with measures to improve child healthcare and when the screened diseases meet criteria such as severe harm, absence of early specific symptoms, being preventable and treatable, and having established therapeutic protocols and medications can it genuinely reduce infant mortality and promote health equity.

With advancements in genomic sequencing technologies, the cost of genetic testing continues to decline. The results of disease biomarker testing serve as corroborative evidence for genetic findings, facilitating more straightforward report interpretation and shortening the turnaround time. Moreover, the results of genetic screening contribute to reducing both false-positive and false-negative rates in NBS and enable rapid disease subtyping and differential diagnosis [55, 56]. The combination of both approaches allows for faster and more accurate disease diagnosis, achieving the goal of early treatment. This is particularly critical for IMD with acute onset in the early neonatal stage, as it enables targeted, rapid, and effective interventions, thereby saving lives and reducing the occurrence of severe complications such as neurological disorders. Most importantly, large-scale population screening can be effectively implemented only through integration with existing, well-established NBS systems.

## Methods of combined screening

### Principles for the combined screening of disease and gene selection, and associated diseases and genes

The general principles for disease selection in combined screening include the following: (1) diseases must have biomarkers suitable for screening; (2) there should be a clear gene–biomarker–disease association; (3) pathogenic variants can be reliably detected on available genomic sequencing platforms and bioinformatics workflows; (4) the age of onset or treatable age should be less than 5 years; (5) the disease should have a high incidence or mortality rate; (6) there should be effective treatments or interventions that significantly alter disease progression; (7) consideration of the sensitivity and specificity of biomarkers and genetic testing methods; and (8) a favorable cost‒benefit ratio for the combined screening program.

General principles for gene selection in combined screening [57, 58] include the following: (1) genes associated with routine NBS conditions, such as *G6PD*, *PAH*, and *PTS*; (2) genes linked to diseases screened by MS/MS or other methods in newborns, including *MMACHC*, *MMUT*, *SLC25A13*, *SLC22A5*, and *GCDH*, which have been confirmed as common pathogenic variants in the Chinese population; (3) clear gene‒disease relationships, as defined by the Clinical Genome Resource (ClinGen) Center of the National Institutes of Health in the United States; (4) the inheritance pattern of genes is definite; and (5) maximizing coverage of clinically common monogenic disorders while balancing technical sensitivity and fully considering the cost-effectiveness of the screening program.

On the basis of these principles and considering diseases already included in national or regional NBS programs (via DBS or urine filter paper tests), through expert consensus, the recommended target diseases for combined screening include 154 disease-causing genes covering 67 IMDs, specifically organic acid metabolism disorders, amino acid metabolism disorders, urea cycle disorders, fatty acid β-oxidation disorders, carnitine transport disorders, creatine synthesis and transport disorders, peroxisomal diseases, and lysosomal storage diseases. The detailed diseases and traditional screening methods used are shown in Tables 1 and 2. Individual laboratories may adjust corresponding combined screening methods according to their technical capabilities and local disease prevalence.

**Table 1:** Diseases recommended for combined biomarker and genetic screening

Number	Disease	Classification	Gene	Inheritance pattern	Specific biomarkers—blood	Specific biomarkers—urine
Organic acid metabolism disorders						
1	Methylmalonic acidemia	Type Mut	*MMUT*	AR	C3, C3/C2, methylmalonic acid, methylcitric acid	Methylmalonic acid, methylcitric acid
		Type A	*MMAA*	AR		Methylmalonic acid, methylcitric acid
		Type B	*MMAB*	AR		Methylmalonic acid, methylcitric acid
		Type CblC	*MMACHC*	AR	C3, C3/C2, homocysteine	Methylmalonic acid, methylcitric acid, homocysteine
		Type CblD	*MMADHC*	AR		Methylmalonic acid, methylcitric acid, homocysteine
		Type CblF	*LMBRD1*	AR		Methylmalonic acid, methylcitric acid, homocysteine
		Type CblJ	*ABCD4*	AR		–
		Type CblX	*HCFC1*	XLR		
2	Propionic acidemia		*PCCA*	AR	C3, C3/C2, methylcitric acid	Methylcitric acid, 3-hydroxypropionic acid, propionylglycine
			*PCCB*	AR		
3	Combined malonic acid and methylmalonic aciduria		*ACSF3*	AR	–	Methylmalonic acid, methylcitric acid, malonic acid
4	Malonyl-CoA decarboxylase deficiency		*MLYCD*	AR	C3DC	Malonic acid
* 5*	Isobutyrylglycinuria		*ACAD8*	AR	C4	–
6	Ethylmalonic encephalopathy		*ETHE1*	AR	C4, C5	–
7	β-Ketothiolase deficiency		*ACAT1*	AR	C4OH, C5:1, C5OH	–
8	Isovaleric acidemia		*IVD*	AR	C5	Isovalerylglycine, 3-hydroxyisovaleric acid
9	2-Methylbutyrylglycinuria		*ACADSB*	AR	C5	–
10	2-Methyl-3-hydroxybutyric aciduria		*HSD17B10*	AR	C5:1, C5OH	–
			*HADH2*	AR		
11	Glutaric acidemia	Type I	*GCDH*	AR	C5DC	Glutaric acid, 3-hydroxyglutaric acid
		Type III	*SUGCT*	AR		–
12	Multiple carboxylase deficiency	Holocarboxylase synthetase deficiency	*HLCS*	AR	C5OH, C3, C3/C2	–
		Biotinidase deficiency	*BTD*	AR	Biotinidase activity (L)	–
13	3-Hydroxy-3-methylglutaracidemia		*HMGCL*	AR	C5OH	–
			*HMGCS2*	AR		
14	3-Methylcrotonyl-CoA carboxylase deficiency		*MCCC1*	AR	C5OH	3-Methylcrotonylglycine, 3-hydroxyisovaleric acid
			*MCCC2*	AR		
15	3-Methylglutaconic acidemia	Type I	*AUH*	AR	C5OH	–
		Type II	*TAFAZZIN*	XLR		
			*TAZ*	XLR		
		Type III	*OPA3*	AR		
		Type V	*DNAJC19*	AR		
		Type VI	*SERAC1*	AR		
		Type VII A/B	*CLPB*	AR		
		Type VIII	*HTRA2*	AR		
		Type IX	*TIMM50*	AR		
16	Alcaptonuria		*HGD*	AR	–	Homogentisic acid
Amino acid metabolism disorders						
17	Maple syrup urine disease		*BCKDHA*	AR	Leu, Ile, Val	–
			*BCKDHB*	AR		
			*DBT*	AR		
			*DLD*	AR		
18	Tyrosinemia	Type I	*FAH*	AR	Tyr, SA	–
		Type II	*TAT*	AR	Tyr	–
		Type III	*HPD*	AR	Tyr	–
19	Hyperphenylalaninemia	Phenylalanine hydroxylase deficiency	*PAH*	AR	Phe, Phe/Tyr	–
		Pterin-4a-carbinolamine dehydratase deficiency	*PCBD1*	AR		
		6-Pyruvoyl-tetrahydrobiopterin synthase deficiency	*PTS*	AR		
		Dihydropteridine reductase deficiency	*QDPR*	AR		
		Guanosine triphosphate cyclohydrolase deficiency	*GCH1*	AR		
		Sepiapterin reductase deficiency	*SPR*	AR		
20	Methionine cycle disorder	Homocysteinemia	*CBS*	AR	Met, homocysteine	Homocysteine
			*MTHFR*	AR	Met (L), homocysteine	
			*MTRR*	AR	Homocysteine	
			*MTR*	AR	Homocysteine	
		Methionine adenosyltransferase I/III deficiency	*MAT1A*	AR/AD	Met	–
		Glycine N-methyltransferase deficiency	*GNMT*	AR		
		S-adenosylhomocysteine hydrolase deficiency	*AHCY*	AR		
		Adenosine kinase deficiency	*ADK*	AR		
21	Hyperprolinemia	Type I	*PRODH*	AR	Pro	–
		Type II	*ALDH4A1*	AR		
22	Non-ketotic hyperglycinemia		*GLDC*	AR	Gly	–
			*AMT*	AR		
			*GCSH*	AR		
23	Hyperlysinemia		*AASS*	AR	Lys	–
24	Cystinuria		*SLC3A1*	AR	–	Cys, Orn, Lys, Arg
			*SLC7A9*	AR		
25	Glutathione synthetase deficiency		*GSS*	AR	5-Oxoproline	–
26	Aromatic L-amino acid decarboxylase deficiency		*DDC*	AR	3-O-methyldopa	–
Urea cycle disorders						
27	Argininemia		*ARG1*	AR	Arg	Arg
28	Argininosuccinic acidemia		*ASL*	AR	Cit, ASA	ASA
29	Carbamoyl phosphate synthetase I deficiency		*CPS1*	AR	Cit (L), Gln	–
30	N-acetylglutamate synthase deficiency		*NAGS*	AR	Cit (L), Gln	–
31	Ornithine transcarbamylase deficiency		*OTC*	XLR	Cit (L), Gln	–
32	Carbonic anhydrase VA deficiency		*CA5A*	AR	Cit (L), Gln	–
33	Argininosuccinate synthetase deficiency		*ASS1*	AR	Cit	Cit
34	Citrin deficiency		*SLC25A13*	AR	Cit, Met, Arg, Phe	Cit
35	Hyperornithinemia–hyperammonemia–homocitrullinemia syndrome		*SLC25A15*	AR	Orn	Orotic acid, uracil
36	Ornithine aminotransferase deficiency		*OAT*	AR	Orn	–
Fatty acid β-oxidation disorders						
37	2, 4-Dienoyl-CoA reductase deficiency		*NADK2*	AR	C10:2	–
38	Very-long-chain acyl-CoA dehydrogenase deficiency		*ACADVL*	AR	C14:1, C14:2, C14, C12:1, C12	–
39	Isolated and trifunctional protein deficiency		*HADHA*	AR	C14OH, C14:1OH, C16OH, C16:1OH, C18OH, C18:1OH	–
			*HADHB*	AR		
40	Medium chain acyl-CoA dehydrogenase deficiency		*ACADM*	AR	C6, C8, C10, C8/C10	–
41	Short-chain acyl-CoA dehydrogenase deficiency		*ACADS*	AR	C4	–
42	Short chain 3-hydroxyacyl-CoA dehydrogenase deficiency		*HADH*	AR	C4OH	–
43	Multiple acyl-CoA dehydrogenase deficiency		*ETFA*	AR	C4–C18	–
			*ETFB*	AR		
			*ETFDH*	AR		
44	Medium chain ketoyl-CoA thiolase deficiency				C6OH, C8, C10OH	–
Primary carnitine deficiency						
45	Primary carnitine deficiency		*SLC22A5*	AR	C0 (L), C2 (L), C3 (L)	–
46	Carnitine palmitoyl transferase II deficiency		*CPT2*	AR	C0 (L), C16, C18, C0/(C16 + 18) (L)	
	-					
47	Carnitine palmitoyl transferase I deficiency		*CPT1A*	AR	C0, C16 (L), C18 (L), C0/(C16 + 18)	–
48	Carnitine-acylcarnitine translocase deficiency		*SLC25A20*	AR	C0 (L), C16, C18, C0/(C16 + 18)	–
Peroxisomal disorders						
49	Adrenoleukodystrophy		*ABCD1*	XLR	C26:0 Lysophosphatidylcholine	–
50	Zellweger syndrome		*PEX1*	AR	C26:0 Lysophosphatidylcholine	–
			*PEX10*	AR		
			*PEX11*	AR		
			*PEX12*	AR		
			*PEX13*	AR		
			*PEX14*	AR		
			*PEX16*	AR		
			*PEX19*	AR		
			*PEX2*	AR		
			*PEX26*	AR		
			*PEX3*	AR		
			*PEX5*	AR		
			*PEX6*	AR		
Creatine synthesis and transport disorders						
51	Guanidinoacetate methyltransferase deficiency		*GAMT*	AR	Guanidinoacetic acid, creatinine (L), guanidinoacetic acid/creatinine	Creatinine (L), guanidinoacetic acid
52	Arginine:glycine transaminase deficiency		*GATM*	AR	–	Creatinine (L), guanidinoacetic acid
53	Creatine transporter deficiency		*SLC6A8*	XLR	–	Creatinine
Lysosomal storage disorders						
54	Mucopolysaccharidosis	Type I	*IDUA*	AR	α-L-iduronidase (L), mucopolysaccharide	–
		Type II	*IDS*	XLR	Iduronate-2-sulfatase (L), mucopolysaccharide	
		Type IIIB	*NAGLU*	AR	α-N-acetylglucosaminidase (L)	
		Type IVA	*GALNS*	AR	N-acetyl-galactosamine-6-sulfatase (L), mucopolysaccharide	
		Type VI	*ARSB*	AR	N-acetyl-galactosamine-4-sulfatase (L)	
55	Krabbe disease		*GALC*	AR	β-Galactocerebrosidase (L)	–
56	Gaucher disease		*GBA*	AR	Acid-β-glucocerebrosidase (L)	–
57	Fabry disease		*GLA*	XLD	α-Galactosidase A (L)	–
58	Niemann–Pick disease type A/B		*SMPD1*	AR	Acid sphingomyelinase (L)	–
59	Pompe disease		*GAA*	AR	Acid α-glucosidase (L)	–
Others						
60	Pyruvate carboxylase deficiency		*PC*	AR	Cit	–
61	Congenital adrenal hyperplasia		*CYP21A2*	AR	17-Hydroxyprogesterone	–
			*CYP11B1*	AR		
62	Galactosemia	Galactokinase deficiency	*GALK1*	AR	Total galactose	–
		Galactose isomerase deficiency	*GALE*	AR		–
		Galactose mutarotase deficiency	*GALM*	AR		–
		Classical galactosemia	*GALT*	AR	Total galactose, galactose-1-phosphate uridyltransferase (L)	–
63	Glucose-6-phosphate dehydrogenase deficiency		*G6PD*	XLD	G6PD (L)	–
64	Glutamate formylaminotransferase deficiency		*FTCD*	AR	Formiminoglutamic acid, C4	–
65	Congenital hypothyroidism		*PAX8*	AR	Thyroid stimulating hormone, thyroxine (L)	
			*THRA*	AR		
			*THRB*	AR		
			*TSHB*	AR		
			*TSHR*	AR		
			*TG*	AR		
			*TPO*	AR		
			*DUOXA2*	AR		
			*DUOX2*	AR		
66	Severe combined immunodeficiency caused by adenosine deaminase deficiency		*ADA*	AR	Adenosine, 2’-deoxyadenosine	
67	Duchenne muscular dystrophy		*DMD*	XLD	Creatine kinase	

*AR* autosomal recessive, *AD* autosomal dominant, *XLR* X-linked recessive, *XLD* X-linked dominant, *C0* carnitine, *C2* acetylcarnitine, *C3* propionylcarnitine, *C3DC* malonylcarnitine, *C4* butyrylcarnitine, *C4OH* 3-hydroxy-butyryl carnitine, *C5* isovaleryl carnitine, *C5:1* tiglyl carnitine, *C5OH* 3-hydroxy-isovaleryl carnitine, *C5DC* glutaryl carnitine, *C6* hexanoyl carnitine,* C6OH* 3-hydroxy-hexanoyl carnitine, *C8* octanoyl carnitine, *C10* decanoyl carnitine, *C10OH* 3-hydroxy-decanoyl carnitine, *C12* dodecanoyl carnitine, *C14* tetradecanoyl carnitine, *C14:2* tetradecadienoyl carnitine, *C16* palmitoyl carnitine, *C18* stearoyl carnitine, *C18:1* oleyl carnitine, *C26:0* hexacosanoyl carnitine, *C14OH* 3-hydroxy myristoyl carnitine, *C16OH* 3-hydroxy palmitoyl carnitine, *C16:1OH* 3-hydroxy palmitoleyl carnitine, *C18:1OH* 3-hydroxy olely carnitine, *SA* succinylacetone, *ASA* argininosuccinic acid, *G6PD* glucose-6-phosphate dehydrogenase

**Table 2:** Summary of the traditional methods used currently

Diseases	Screening methods
Organic acid metabolism disorders	MS/MS, colorimetric assay, fluorometric assay
Amino acid metabolism disorders	MS/MS, fluorometric assay
Urea cycle disorders	MS/MS
Fatty acid β-oxidation disorders	MS/MS
Primary carnitine deficiency	MS/MS
Peroxisomal disorders	MS/MS
Creatine synthesis and transport disorders	MS/MS
Lysosomal storage disorders	MS/MS, fluorometric assay
Others	
Pyruvate carboxylase deficiency	MS/MS
Congenital adrenal hyperplasia	Fluorometric assay
Galactosemia	Paigen test, colorimetric assay, fluorometric assay
Glucose-6-phosphate dehydrogenase deficiency	Fluorometric assay
Glutamate formylaminotransferase deficiency	MS/MS
Congenital hypothyroidism	Fluorometric assay, ELISA
Severe combined immunodeficiency caused by adenosine deaminase deficiency	MS/MS
Duchenne muscular dystrophy	MS/MS, ELISA, fluorometric assay

*MS/MS* includes flow-injection tandem mass spectrometry, liquid chromatography tandem mass spectrometry, *ELISA* enzyme linked immunosorbent assay

### Informed consent, sample collection, detection and reporting for combined screening

#### Informed consent

Nurses should provide comprehensive education on relevant knowledge to the guardians of neonates and truthfully inform the guardians of all key information regarding the combined screening, including target diseases covered in the screening, screening techniques and their limitations, costs, general procedures, sample collection methods, the resulting inquiry process, potential risks, etc. Based on full disclosure and adherence to the principle of informed choice, a written informed consent form should be signed with the guardian to clarify the rights and obligations of both parties during the screening process to ensure the smooth implementation of screening and safeguard the legitimate rights and interests of both parties.

#### Sample collection, detection and reporting

##### Preparation of the sample collection card

It is necessary to fill in the blood collection cards carefully, ensuring clear handwriting and complete registration. The newborn common genetic and metabolic disease screening blood collection cards with legible handwriting and complete information, including basic information, such as name, sex, referring hospital, date of birth, and blood collection date of the neonate, were accurately collected. Moreover, relevant information, such as the name, contact number, contact address of the guardian, and blood collector, was collected. The barcode at the designated position on the sample should be affixed properly to avoid sample confusion effectively and ensure that each sample can be accurately identified and tracked.

##### Sample collection

The collection of heel blood should be strictly controlled within 2–7 days after the birth of the newborn, no later than 20 day postpartum. For low-birthweight infants (< 1800 g) and preterm infants (< 37 gestational weeks), blood sample collection is repeated in the first month of life at 15 and 30 days [59]. Two additional 8-mm diameter blood spots are needed beyond routine screening to meet combined testing needs. After the blood samples are collected, they should be allowed to dry naturally at room temperature for more than 4 hours to make DBSs on filter paper. During the drying process, placing the samples under an electric hair dryer near a heating radiator, on an electric stove, in sunlight or under a strong light source for baking, or stacking them together, is strictly prohibited. Moreover, contact between the samples and other surfaces should be avoided to prevent the samples from being contaminated or damaged. The qualified DBSs on filter paper should be packaged independently in a moisture proof manner, sealed, and then temporarily stored in a refrigerator at 2–8 ℃. They should be promptly delivered to the designated laboratory in an environment of less than 25 ℃ for testing to ensure the quality and stability of the samples.

##### Sample detection and reporting

The detection laboratory should be equipped with advanced detection equipment, including time-resolved fluorescence analysis, MS/MS analysis, liquid phase capture sequencing, and NGS, along with robust bioinformatics analysis capabilities. Both biomarker detection reports and genetic testing reports should be issued to relevant personnel simultaneously within 15 working days after sample collection to provide a basis for subsequent diagnosis and treatment in a timely manner.

Additional information in the report should include interpretation and annotations of results, limitations of the testing methodology and the clinical significance of the findings.

##### Handling special cases

For IMDs with significantly abnormal biomarkers and early onset manifestations in the neonatal period, treatment should be initiated immediately to avoid missing the critical therapeutic window. During the treatment process, upon receiving genetic detection results, a more precise and comprehensive treatment plan should be further formulated according to the detailed genetic findings.

#### Genetic screening testing protocol

For the genetic testing component of combined screening, it is recommended to employ NGS-based targeted capture techniques (including liquid‒phase hybridization capture or multiplex PCR enrichment) to analyze the exon4ic regions and flanking sequences of the target genes, with coverage extended to known pathogenic or likely pathogenic (P/LP) variants in non-coding regions. Quality control of the sequencing data should adhere to standard NGS guidelines, ensuring that the coverage depth of the target area is greater than 300 × , that the depth of more than 99.5% of the target sequence area reaches more than 20 × and that the depth of more than 99% of the target sequence area reaches more than 100 × . In the secondary analysis pipeline, the use of GATK or other industry-validated analytical tools for sequencing data analysis is recommended, with the reference genome options being GRCh37/hg19 or GRCh38/hg38. For tertiary analysis, annotation should be performed via software, such as variant effect predictor or ANNOVAR, followed by filtering of known pathogenic variants on the basis of major databases, including ClinVar and the Human Gene Mutation Database, which curates known or suspected disease-causing variants. As supplementary evidence for the assessment of variant pathogenicity, bioinformatics software can be employed to predict the impact of variants: REVEL or CADD for missense variant prediction and SpliceAI or dbscSNV for evaluating potential splicing effects. All sequence variants should be named according to the Human Genome Variation Society nomenclature standards, and each variant should be classified for pathogenicity in accordance with the variant interpretation guidelines of the American College of Medical Genetics and Genomics and the ClinGen of the National Institutes of Health.

Deficiency of 21-hydroxylase (21-OHD), which causes congenital adrenal hyperplasia, is attributed to mutations in the *CYP21A2* gene. This gene has a highly homologous pseudogene, *CYP21A1P*, with 98% and 95% sequence identity in exons and introns, respectively. Among pathogenic mutations in 21-OHD, 95% arise from recombination events between the functional gene and pseudogene: approximately 75% due to mitotic gene conversion, where variants in *CYP21A1P* are transferred to *CYP21A2*, and approximately 20%–25% caused by meiotic non-allelic homologous recombination. Owing to the limited read length of conventional NGS, misalignment may occur when highly homologous regions are analyzed. For *CYP21A2* gene mutation screening, we recommend the use of either PCR-based NGS or long-read sequencing technologies, followed by the use of multiplex ligation-dependent probe amplification combined with Sanger sequencing for further validation [60].

Citrin deficiency, caused by pathogenic variants in *SLC25A13*, is frequently associated with complex structural variations, including IVS4ins6kb and IVS16ins3kb. NGS and specific bioinformatics algorithms are employed to screen for mutations in the *SLC25A13* gene. When necessary, long-range PCR was used for further detection.

#### Principles for the determination and handling of combined screening results

A positive combined screening result refers to the discovery of P/LP variants that are highly correlated with clinical (early onset cases) and biomarkers and have a matching genetic pattern: (1) autosomal inheritance: in the dominant inheritance pattern, a heterozygous P/LP variant is present for the disease indicated by the biomarkers in the genetic screening; in the recessive inheritance pattern, a biallelic P/LP variant is present for the disease indicated by the biomarkers in the genetic screening and (2) X-linked inheritance: for diseases indicated by biomarkers in genetic screening, there is a P/LP variation in the gene on the X chromosome. In the case of dominant inheritance patterns, all patients carrying the mutation will be affected; in recessive inheritance patterns, female homozygotes and male hemizygotes are affected, whereas female heterozygotes are usually normal; in incomplete dominant inheritance patterns, female homozygotes and male hemizygotes are affected, and female heterozygotes can have a clinical manifestation spectrum ranging from completely asymptomatic to severely symptomatic [61]. This phenomenon occurs, because skewed X chromosome inactivation (XCI) leads to preferential inactivation of the X chromosome carrying the wild-type allele [62], resulting in a greater proportion of mutant cells than wild-type cells [63, 64], which in turn causes clinical symptoms in female heterozygotes. In some affected females, the proportion of mutant cells is greater than 95%, indicating a severe skew in XCI [65]. In this genetic pattern, XCI is the main factor determining the severity of clinical involvement in female heterozygotes [66]. Fabry disease and ornithine transcarbamylase deficiency both follow this genetic pattern [66, 67].

The following three situations require further confirmation: (1) normal characteristic indicators but genetic testing has identified P/LP variations that conform to genetic patterns (such as neonatal intrahepatic cholestasis caused by citrin deficiency, multiple acyl-CoA dehydrogenase deficiency and other diseases); (2) markedly abnormal characteristic indicators but no P/LP variants identified that align with the clinical phenotype and expected genetic mode in genetic testing; and (3) genetic testing has identified variants that conform to the genetic pattern, among which there is at least one VUS.

For the above situations, family verification and/or other genetic sequencing techniques should be carried out, combined with urine organic acid analysis, enzyme activity determination, very long-chain fatty acid determination, amino acid determination, blood ammonia detection, liver function tests, etc., to rule out maternal genetic metabolic disorders as well as the influences of nutrition, diseases and drugs on the infant and mother. A definite diagnosis should be made through comprehensive analysis. If there are already clinical manifestations and/or metabolic disorders, the infant should be immediately admitted to the hospital for treatment; if there are no clinical manifestations and the metabolism is relatively stable, outpatient treatment can be adopted, with regular reexaminations, follow-ups and management.

A negative combined screening indicates that the newborn shows no clinical symptoms during the neonatal period, has normal disease biomarkers, and that no P/LP variants consistent with the genetic pattern are identified through genetic screening. In such cases, no follow-up is needed.

### Special circumstances of heterozygous variants in autosomal recessive inherited metabolic disorders

In autosomal recessive inherited metabolic disorders (ARIMDs), a pathogenic variant at a single locus in certain diseases can lead to partial deficiency of enzyme function, causing mild abnormalities in characteristic markers.

For multimeric proteins, variant proteins can disrupt the formation, function and stability of homomeric or heteromeric multimers through dominant-negative effects or interfere with the interactions between wild-type proteins and other molecules, thereby impairing the partial function of wild-type proteins [68]. For example, citrullinemia type 1 (CTLN1) is recessively inherited and caused by variants in the *ASS1* gene, which encodes argininosuccinate synthase (ASS). The crystal structure of human ASS is a functional tetramer consisting of two identical dimers. The most common variant, p.R363W, likely affects the oligomerization of the protein via a dominant‐negative effect, which affects the other normal counter partner. This caused an increase in citrulline levels in carriers [69–71]. The heteromeric multimeric enzyme methylcrotonyl-CoA carboxylase is a heteropolymer composed of MCCCα and MCCCβ subunits (encoded by *MCCC1* and *MCCC2*, respectively). Mutations at specific loci in either *MCCC1* or *MCCC2* can exert a dominant-negative effect: the mutant protein product interferes with the normal function of the wild-type MCC complex, resulting in suppressed MCC enzyme activity. This functional impairment further leads to mildly elevated levels of metabolites associated with 3-methylcrotonyl-CoA carboxylase deficiency in heterozygotes [72].

The impact of the dominant negative effect varies, and sometimes, it can even lead to significant functional impairment of the protein. The c.791G > A (R264H) mutation site of the *MAT1A* gene in hypermethioninemia alters the residue position at the dimer interface, preventing substrate binding and resulting in loss of activity and causing disease. This alteration at the site is dominant inheritance. c.769G > A (p.G257R) is located in a critical structural domain of the protein. This variant may affect the dimeric interaction of the protein. It has been reported in patients with hypermethioninemia in homozygous, compound heterozygous and single heterozygous forms, suggesting that it may exist in both dominant and recessive modes [73]. These two variants generally do not significantly increase the methionine content and often do not require special treatment. However, they may be detected via biomarker-based screening or newborn genetic screening, necessitating regular monitoring of methionine levels.

In general, patients with heterozygous variations in ARIMD typically do not exhibit clinical symptoms. However, whether regular monitoring and management are necessary for these individuals remains to be further studied.

### Establishment of a combined screening system

Only when the combined screening system is closely integrated with measures to improve children’s healthcare and becomes a public health measure for obtaining high-quality medical services can it tangibly improve children’s health. Accordingly, genetic screening should be incorporated into existing biomarker-based screening systems.

Within the existing management system, provincial/municipal NBS centers should establish integrated platforms for biomarker and genetic testing. Within 48 hours after the birth of an infant, a series of tasks, including obtaining informed consent, sample collection, delivery, testing, diagnosis, treatment, follow-up assessment, and comprehensive management, should be carried out simultaneously with the screening of biomarkers. The personnel structure of combined screening should cover individuals with professional backgrounds in laboratory science, molecular genetics, bioinformatics, clinical medicine, etc. All personnel must strictly follow the duties and technical procedures for diagnosis and treatment as stipulated in relevant regulations. The quality control center conducts quality control to determine the concentration of biomarkers, DNA extraction, library preparation, amplification, etc., to ensure the accuracy and reliability of the test results. A complete data analysis and information management system is established to provide a standardized communication mechanism for traditional NBS laboratories and newborn genetic screening laboratories, ensuring that results and data can be transmitted accurately and promptly. At the same time, a combined screening team composed of testing personnel, genetic counselors, and clinical physicians should be formed to establish diagnosis and preliminary treatment plans.

**Fig. 1:**
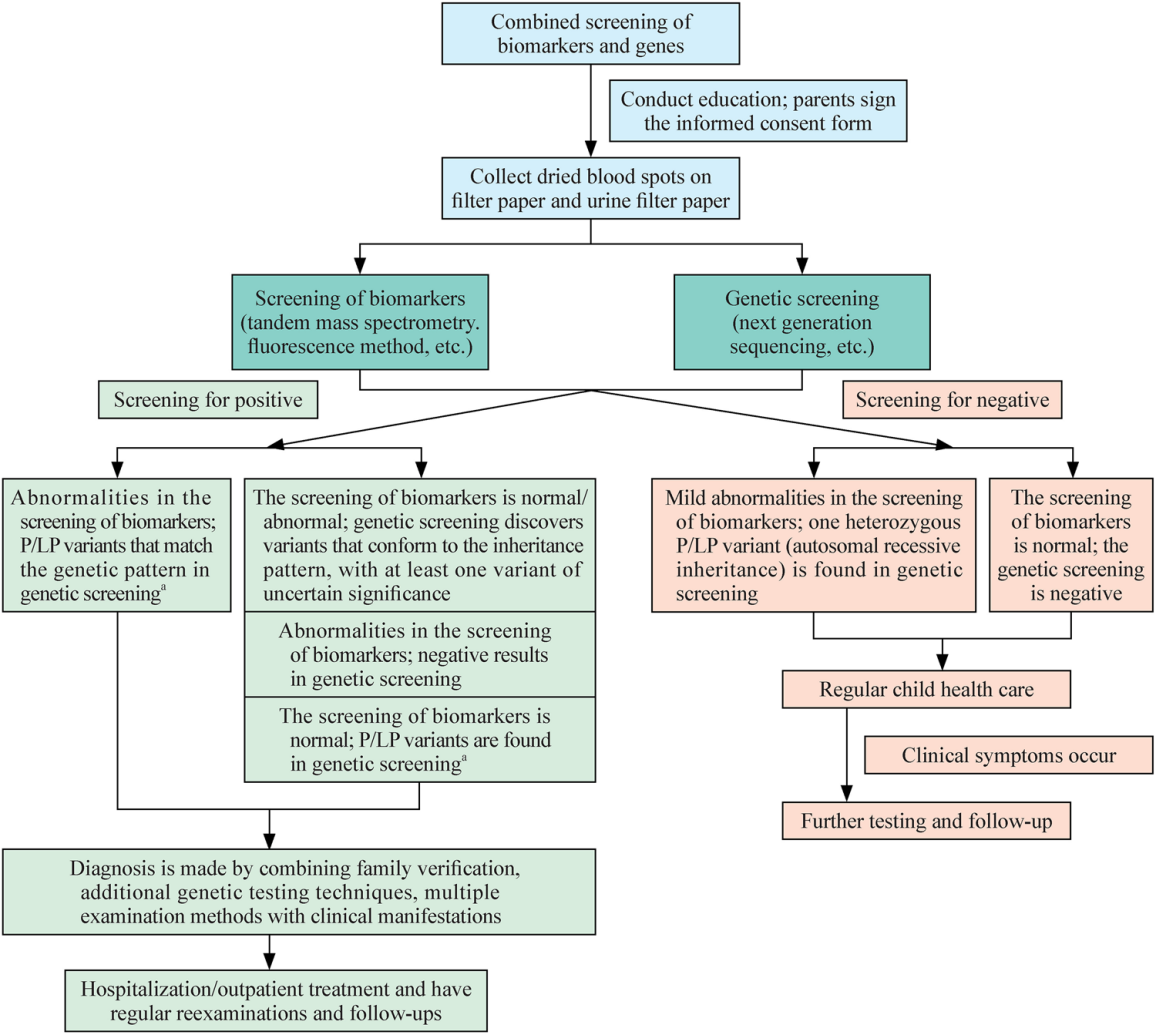
Diagnosis and treatment pathway for combined screening of biomarkers and genes. *P/LP* pathogenic/likely pathogenic. ^a^AD inheritance: one heterozygous P/LP variant; AR inheritance: biallelic P/LP variants; X-linked recessive inheritance: female biallelic or male hemizygous P/LP variants; X-linked dominant or incomplete dominant inheritance: female heterozygous or male hemizygous P/LP variants; the dominant mutation locus of the AR genetic disease MATD: *MAT1A* Arg264His (R264H)

In conclusion, this expert consensus defines a comprehensive framework for neonatal combined genetic and biomarker screening, outlining clear disease/gene selection criteria, standardized operational procedures, and specific strategies for managing special cases (Fig. 1). While this approach holds great promise for improving infant health outcomes, its widespread implementation faces technical, ethical, and validation challenges. Future efforts must focus on optimizing cost-effectiveness, improving data interpretation, and establishing a standardized national system. Integrating this screening into established newborn screening programs will ultimately reduce the burden of treatable genetic diseases and advance health equity.

## Data Availability

Not required for this article.

## References

[1] Wiley V, Webster D, Loeber G. Screening pathways through China, the Asia Pacific region, the world. Int J Neonatal Screen. 2019;5:26. 33072985 10.3390/ijns5030026 PMC7510188

[2] Octavius GS, Daleni VA, Sagala YDS. An insight into Indonesia’s challenges in implementing newborn screening programs and their future implications. Children. 2023;10:1216. 37508713 10.3390/children10071216 PMC10378005

[3] Gu XF, Han LS, Yang YG. Screening status and prospect of neonatal genetic metabolic diseases in China. Rare Dis Res. 2015;1:15–22 (**in Chinese**).

[4] Luo X, Sun Y, Xu F, Guo J, Li L, Lin Z, et al. A pilot study of expanded newborn screening for 573 genes related to severe inherited disorders in China: results from 1,127 newborns. Ann Transl Med. 2020;8:1058. 33145277 10.21037/atm-20-1147 PMC7575988

[5] Pereira S, Robinson JO, Gutierrez AM, Petersen DK, Hsu RL, Lee CH, et al. Perceived benefits, risks, and utility of newborn genomic sequencing in the BabySeq project. Pediatrics. 2019;143(Suppl 1):6–13. 10.1542/peds.2018-1099C PMC6480393 30600265

[6] Hao C, Guo R, Hu X, Qi Z, Guo Q, Liu X, et al. Newborn screening with targeted sequencing: a multicenter investigation and a pilot clinical study in China. J Genet Genom. 2021;49:13–9. 10.1016/j.jgg.2021.08.008 34474183

[7] Yang RL, Qian GL, Wu DW, Miao JK, Yang X, Wu BQ, et al. A multicenter prospective study of next-generation sequencing-based newborn screening for monogenic genetic diseases in China. World J Pediatr. 2023;19:663–73. 36847978 10.1007/s12519-022-00670-x PMC10258179

[8] Chen T, Fan C, Huang Y, Feng J, Zhang Y, Miao J, et al. Genomic sequencing as a first-tier screening test and outcomes of newborn screening. JAMA Netw Open. 2023;6:e2331162. 37656460 10.1001/jamanetworkopen.2023.31162 PMC10474521

[9] Wang X, Xia Z, He Y, Zhou X, Zhang H, Gao C, et al. Newborn screening for G6PD deficiency in Xiamen, China: prevalence, variant spectrum, and genotype-phenotype correlations. Front Genet. 2021;12:718503. 34659341 10.3389/fgene.2021.718503 PMC8517332

[10] Huang LW, Huang ZZ, Yan JB, Zhang Y, Shi YZ, Zhu SS, et al. Effects of delivery and storage conditions on the concentration of amino acids and carnitine in neonatal dry blood spot specimens. Zhejiang Da Xue Xue Bao Yi Xue Ban. 2019;49:565–73 (**in Chinese**). 10.3785/j.issn.1008-9292.2020.10.03 PMC8800740 33210481

[11] Winter T, Lange A, Hannemann A, Nauck M, Müller C. Contamination of dried blood spots - an underestimated risk in newborn screening. Clin Chem Lab Med. 2018;56:278–84. 28763295 10.1515/cclm-2017-0270

[12] George RS, Moat SJ. Effect of dried blood spot quality on newborn screening analyte concentrations and recommendations for minimum acceptance criteria for sample analysis. Clin Chem. 2016;62:466–75. 26647314 10.1373/clinchem.2015.247668

[13] Holub M, Tuschl K, Ratschmann R, Strnadová KA, Mühl A, Heinze G, et al. Influence of hematocrit and localization of punch in dried blood spots on levels of amino acids and acylcarnitines measured by tandem mass spectrometry. Clin Chim Acta. 2006;373:27–31. 16797519 10.1016/j.cca.2006.04.013

[14] Li XL, Shen WQ, Xu XW. Factors affecting amino acid levels in dry blood filter paper by liquid-mass tandem mass spectrometry. J Clin Lab. 2009;27:302–4 (**in Chinese**).

[15] McHugh D, Cameron CA, Abdenur JE, Abdulrahman M, Adair O, Al Nuaimi SA, et al. Clinical validation of cutoff target ranges in newborn screening of metabolic disorders by tandem mass spectrometry: a worldwide collaborative project. Genet Med. 2011;13:230–54. 21325949 10.1097/GIM.0b013e31820d5e67

[16] Huang ZZ, Yan JB, Shang SQ. Preliminary application of Region 4 Stork system in tandem mass spectrometry screening of neonatal genetic metabolic diseases. Chin J Lab Med. 2018;41:300–4 (**in Chinese**).

[17] Zhou W, Li H, Wang C, Wang X, Gu M. Newborn screening for methylmalonic acidemia in a Chinese population: molecular genetic confirmation and genotype phenotype correlations. Front Genet. 2019;9:726. 30728829 10.3389/fgene.2018.00726 PMC6351470

[18] Suormala T, Wick H, Baumgartner ER. Low biotinidase activity in plasma of some preterm infants: possible source of false-positive screening results. Eur J Pediatr. 1988;147:478–80. 3409923 10.1007/BF00441970

[19] Valentine CJ, Puthoff TD. Enhancing parenteral nutrition therapy for the neonate. Nutr Clin Pract. 2007;22:183–93. 17374792 10.1177/0115426507022002183

[20] Minoli I, Räihä NC. Effects of two different doses of amino acid supplementation on growth and blood amino acid levels in premature neonates admitted to the neonatal intensive care unit: a randomized, controlled trial. Pediatrics. 2008;121:655–6. 18310223 10.1542/peds.2007-3786

[21] Yang L, Zhang Y, Yang J, Huang X. Effects of birth weight on profiles of dried blood amino-acids and acylcarnitines. Ann Clin Biochem. 2018;55:92–9. 29064274 10.1177/0004563216688038

[22] Turgeon CT, Magera MJ, Cuthbert CD, Loken PR, Gavrilov DK, Tortorelli S, et al. Determination of total homocysteine, methylmalonic acid, and 2-methylcitric acid in dried blood spots by tandem mass spectrometry. Clin Chem. 2010;56:1686–95. 20807894 10.1373/clinchem.2010.148957

[23] Clark RH, Chace DH, Spitzer AR, Pediatrix Amino Acid Study Group. Effects of two different doses of amino acid supplementation on growth and blood amino acid levels in premature neonates admitted to the neonatal intensive care unit: a randomized, controlled trial. Pediatrics. 2007;120:1286–96. 18055678 10.1542/peds.2007-0545

[24] Chace DH, De Jesús VR, Lim TH, Hannon WH, Clark RH, Spitzer AR. Detection of TPN contamination of dried blood spots used in newborn and metabolic screening and its impact on quantitative measurement of amino acids. Clin Chim Acta. 2011;412:1385–90. 21514290 10.1016/j.cca.2011.04.009

[25] Boemer F, Schoos R, de Halleux V, Kalenga M, Debray FG. Surprising causes of C5-carnitine false positive results in newborn screening. Mol Genet Metab. 2014;111:52–4. 24291264 10.1016/j.ymgme.2013.11.005

[26] Browning MF, Levy HL, Wilkins-Haug LE, Larson C, Shih VE. Fetal fatty acid oxidation defects and maternal liver disease in pregnancy. Obstet Gynecol. 2006;107:115–20. 16394048 10.1097/01.AOG.0000191297.47183.bd

[27] Ning Q, Hu XL, Yu YL. Diagnosis and treatment of infant secondary methylmalonic aciduria caused by maternal vitamin B12 deficiency. Chin J Perinat Med. 2005:179–82

[28] Marble M, Copeland S, Khanfar N, Rosenblatt DS. Neonatal vitamin B12 deficiency secondary to maternal subclinical pernicious anemia: identification by expanded newborn screening. J Pediatr. 2008;152:731–3. 18410783 10.1016/j.jpeds.2008.01.023

[29] Ryckman KK, Berberich SL, Shchelochkov OA, Cook DE, Murray JC. Clinical and environmental influences on metabolic biomarkers collected for newborn screening. Clin Biochem. 2013;46:133–8. 23010448 10.1016/j.clinbiochem.2012.09.013 PMC3534803

[30] Ivica J, Adam F, Wortel L, Kalika T, Pelly H, Gauthier J, et al. Development of a second-tier method for C4, C5 and C2 acylcarnitine analysis in plasma. Clin Biochem. 2024;123:110698. 38048898 10.1016/j.clinbiochem.2023.110698

[31] Holtkamp U, Klein J, Sander J, Peter M, Janzen N, Steuerwald U, et al. EDTA in dried blood spots leads to false results in neonatal endocrinologic screening. Clin Chem. 2008;54:602–5. 18310148 10.1373/clinchem.2007.096685

[32] Estrella J, Wilcken B, Carpenter K, Bhattacharya K, Tchan M, Wiley V. Expanded newborn screening in New South Wales: missed cases. J Inherit Metab Dis. 2014;37:881–7. 24970580 10.1007/s10545-014-9727-2

[33] Ko JM, Park KS, Kang Y, Nam SH, Kim Y, Park I, et al. A new integrated newborn screening workflow can provide a shortcut to differential diagnosis and confirmation of inherited metabolic diseases. Yonsei Med J. 2018;59:652–61. 29869463 10.3349/ymj.2018.59.5.652 PMC5990675

[34] Park KJ, Park S, Lee E, Park JH, Park JH, Park HD, et al. A population-based genomic study of inherited metabolic diseases detected through newborn screening. Ann Lab Med. 2016;36:561–72. 27578510 10.3343/alm.2016.36.6.561 PMC5011110

[35] Lin Y, Liu Y, Zhu L, Le K, Shen Y, Yang C, et al. Combining newborn metabolic and genetic screening for neonatal intrahepatic cholestasis caused by citrin deficiency. J Inherit Metab Dis. 2019;43:467–77. 31845334 10.1002/jimd.12206

[36] Wang LY, Chen NI, Chen PW, Chiang SC, Hwu WL, Lee NC, et al. Newborn screening for citrin deficiency and carnitine uptake defect using second-tier molecular tests. BMC Med Genet. 2013;14:24. 23394329 10.1186/1471-2350-14-24 PMC3575349

[37] Maloney B, Park S, Sowizral M, Brackett I, Moslehi R, Chung WK, et al. Factors influencing creatine kinase-MM concentrations in newborns and implications for newborn screening for Duchenne muscular dystrophy. Clin Biochem. 2023;118:110614. 37479106 10.1016/j.clinbiochem.2023.110614

[38] Ghosh S, Albert MH, Hauck F, Hönig M, Schütz C, Schulz A, et al. Newborn screening for severe combined immunodeficiencies (SCID) in Germany. Bundesgesundheitsblatt Gesundheitsforschung Gesundheitsschutz. 2023;66:1222–31 (**in German**). 37726421 10.1007/s00103-023-03773-6 PMC10622353

[39] Dangouloff T, Vrščaj E, Servais L, Osredkar D, SMA NBS World Study Group. Newborn screening programs for spinal muscular atrophy worldwide: where we stand and where to go. Neuromuscul Disord. 2021;31:574–82. 33985857 10.1016/j.nmd.2021.03.007

[40] Tesorero R, Janda J, Hörster F, Feyh P, Mütze U, Hauke J, et al. A high-throughput newborn screening approach for SCID, SMA, and SCD combining multiplex qPCR and tandem mass spectrometry. PLoS ONE. 2023;18:e0283024. 36897914 10.1371/journal.pone.0283024 PMC10004496

[41] Wojcik MH, Zhang T, Ceyhan-Birsoy O, Genetti CA, Lebo MS, Yu TW, et al. Discordant results between conventional newborn screening and genomic sequencing in the BabySeq project. Genet Med. 2021;23:1372–5. 33772220 10.1038/s41436-021-01146-5 PMC8263473

[42] Green RC, Shah N, Genetti CA, Yu T, Zettler B, Uveges MK, et al. Actionability of unanticipated monogenic disease risks in newborn genomic screening: findings from the BabySeq project. Am J Hum Genet. 2023;110:1034–45. 37279760 10.1016/j.ajhg.2023.05.007 PMC10357495

[43] Friedman JM, Cornel MC, Goldenberg AJ, Lister KJ, Sénécal K, Vears DF, et al. Genomic newborn screening: public health policy considerations and recommendations. BMC Med Genomics. 2017;10:9. 28222731 10.1186/s12920-017-0247-4 PMC5320805

[44] Gold NB, Adelson SM, Shah N, Williams S, Bick SL, Zoltick ES, et al. Perspectives of rare disease experts on newborn genome sequencing. JAMA Netw Open. 2023;6:e2312231. 37155167 10.1001/jamanetworkopen.2023.12231 PMC10167563

[45] Roman TS, Crowley SB, Roche MI, Foreman AKM, O’Daniel JM, Seifert BA, et al. Genomic sequencing for newborn screening: results of the NC NEXUS project. Am J Hum Genet. 2020;107:596–611. 32853555 10.1016/j.ajhg.2020.08.001 PMC7536575

[46] Willig LK, Petrikin JE, Smith LD, Saunders CJ, Thiffault I, Miller NA, et al. Whole-genome sequencing for identification of Mendelian disorders in critically ill infants: a retrospective analysis of diagnostic and clinical findings. Lancet Respir Med. 2015;3:377–87. 25937001 10.1016/S2213-2600(15)00139-3 PMC4479194

[47] Lancet T. Genomic newborn screening: current concerns and challenges. Lancet. 2023;402:265. 37481265 10.1016/S0140-6736(23)01513-1

[48] Downie L, Bouffler SE, Amor DJ, Christodoulou J, Yeung A, Horton AE, et al. Gene selection for genomic newborn screening: moving toward consensus? Genet Med. 2024;26:101077. 38275146 10.1016/j.gim.2024.101077

[49] Ceyhan-Birsoy O, Machini K, Lebo MS, Yu TW, Agrawal PB, Parad RB, et al. A curated gene list for reporting results of newborn genomic sequencing. Genet Med. 2017;19:809–18. 28079900 10.1038/gim.2016.193 PMC5507765

[50] Jiang MA. Rapid targeted genomic testing: a powerful tool for diagnostic evaluation of critically ill neonates and infants with suspected genetic diseases. Ann Lab Med. 2023;43:223–4. 36544333 10.3343/alm.2023.43.3.223 PMC9791018

[51] Kingsmore SF, Wright M, Olsen L, Schultz B, Protopsaltis L, Averbuj D, et al. Genome-based newborn screening for severe childhood genetic diseases has high positive predictive value and sensitivity in a NICU pilot trial. Am J Hum Genet. 2024;111:2643–67. 39642868 10.1016/j.ajhg.2024.10.020 PMC11639094

[52] Wang X, Sun Y, Guan XW, Wang YY, Hong DY, Zhang ZL, et al. Newborn genetic screening is highly effective for high-risk infants: a single-centre study in China. J Glob Health. 2023;13:04128. 37824171 10.7189/jogh.13.04128 PMC10569371

[53] Huang X, Wu D, Zhu L, Wang W, Yang R, Yang J, et al. Application of a next-generation sequencing (NGS) panel in newborn screening efficiently identifies inborn disorders of neonates. Orphanet J Rare Dis. 2022;17:66. 35193651 10.1186/s13023-022-02231-x PMC8862216

[54] Maron JL, Kingsmore S, Gelb BD, Vockley J, Wigby K, Bragg J, et al. Rapid whole-genomic sequencing and a targeted neonatal gene panel in infants with a suspected genetic disorder. JAMA. 2023;330:161–9. 37432431 10.1001/jama.2023.9350 PMC10336625

[55] Jiang LH, Yang RL, Dong A, Wu BQ, Zhao ZY. Progress of neonatal screening in China. Zhejiang Da Xue Xue Bao Yi Xue Ban. 2019;52:673–82 (**in Chinese**). 10.3724/zdxbyxb-2023-0467 PMC10764191 38115737

[56] Chen PC, Zhao ZY. Progress of international neonatal disease screening. Chin J Pract Pediatr. 2019;38:72–6 (**in Chinese**).

[57] Zhang WR, Zhao ZY. Research progress of genetic screening for neonatal diseases. Zhonghua Er Ke Za Zhi. 2019;58:1033–7 (**in Chinese**). 10.3760/cma.j.cn112140-20200614-00620 33256331

[58] Neonatal Inherited Metabolic Disease Screening Group, Birth Defects and Control Professional Committee, Chinese Preventive Medicine Association; Neonatology Group, Pediatrics Branch, Chinese Medical Association. Expert consensus on neonatal genetic screening in China: application of high-throughput sequencing in single-gene disease screening. Chin J Pract Pediatr. 2019;38:31–6 (**in Chinese**).

[59] Fecarotta S, Vaccaro L, Verde A, Alagia M, Rossi A, Colantuono C, et al. Combined biochemical profiling and DNA sequencing in the expanded newborn screening for inherited metabolic diseases: the experience in an Italian reference center. Orphanet J Rare Dis. 2025;20:38. 39856690 10.1186/s13023-025-03546-1 PMC11762513

[60] Li H, Zhu X, Yang Y, Wang W, Mao A, Li J, et al. Long-read sequencing: an effective method for genetic analysis of *CYP21A2* variation in congenital adrenal hyperplasia. Clin Chim Acta. 2023;547:117419. 37276943 10.1016/j.cca.2023.117419

[61] Echevarria L, Benistan K, Toussaint A, Dubourg O, Hagege AA, Eladari D, et al. X-chromosome inactivation in female patients with Fabry disease. Clin Genet. 2015;89:44–54. 25974833 10.1111/cge.12613

[62] Muers MR, Sharpe JA, Garrick D, Sloane-Stanley J, Nolan PM, Hacker T, et al. Defining the cause of skewed X-chromosome inactivation in X-linked mental retardation by use of a mouse model. Am J Hum Genet. 2007;80:1138–49. 17503331 10.1086/518369 PMC1867101

[63] Migeon BR. X inactivation, female mosaicism, and sex differences in renal diseases. J Am Soc Nephrol. 2008;19:2052–9. 18448583 10.1681/ASN.2008020198

[64] Viggiano E, Politano L. X chromosome inactivation in carriers of Fabry disease: review and meta-analysis. Int J Mol Sci. 2021;22:7663. 34299283 10.3390/ijms22147663 PMC8304911

[65] Redonnet-Vernhet I, Ploos van Amstel JK, Jansen RP, Wevers RA, Salvayre R, Levade T. Uneven X inactivation in a female monozygotic twin pair with Fabry disease and discordant expression of a novel mutation in the alpha-galactosidase A gene. J Med Genet. 1996;33:682–8. 8863162 10.1136/jmg.33.8.682 PMC1050704

[66] Dobrovolny R, Dvorakova L, Ledvinova J, Magage S, Bultas J, Lubanda JC, et al. Relationship between X-inactivation and clinical involvement in Fabry heterozygotes. Eleven novel mutations in the alpha-galactosidase A gene in the Czech and Slovak population. J Mol Med. 2005;83:647–54. 15806320 10.1007/s00109-005-0656-2

[67] Rahayatri TH, Uchida H, Sasaki K, Shigeta T, Hirata Y, Kanazawa H, et al. Hyperammonemia in ornithine transcarbamylase-deficient recipients following living donor liver transplantation from heterozygous carrier donors. Pediatr Transplant. 2016. 10.1111/petr.12848. 27891735 10.1111/petr.12848

[68] Zschocke J, Byers PH, Wilkie AOM. Mendelian inheritance revisited: dominance and recessiveness in medical genetics. Nat Rev Genet. 2023;24:442–63. 36806206 10.1038/s41576-023-00574-0

[69] Diez-Fernandez C, Rüfenacht V, Häberle J. Mutations in the human argininosuccinate synthetase (*ASS1*) gene, impact on patients, common changes, and structural considerations. Hum Mutat. 2017;38:471–84. 28111830 10.1002/humu.23184

[70] Häberle J, Pauli S, Linnebank M, Kleijer WJ, Bakker HD, Wanders RJ, et al. Structure of the human argininosuccinate synthetase gene and an improved system for molecular diagnostics in patients with classical and mild citrullinemia. Hum Genet. 2002;110:327–33. 11941481 10.1007/s00439-002-0686-6

[71] Chen HA, Hsu RH, Chang KL, Huang YC, Chiang YC, Lee NC, et al. Asymptomatic ASS1 carriers with high blood citrulline levels. Mol Genet Genomic Med. 2022;10:e2007. 35726796 10.1002/mgg3.2007 PMC9482393

[72] Baumgartner MR, Dantas MF, Suormala T, Almashanu S, Giunta C, Friebel D, et al. Isolated 3-methylcrotonyl-CoA carboxylase deficiency: evidence for an allele-specific dominant negative effect and responsiveness to biotin therapy. Am J Hum Genet. 2004;75:790–800. 15359379 10.1086/425181 PMC1182108

[73] Panmanee J, Antonyuk SV, Hasnain SS. Structural basis of the dominant inheritance of hypermethioninemia associated with the Arg264His mutation in the *MAT1A* gene. Acta Crystallogr D Struct Biol. 2020;76:594–607. 32496220 10.1107/S2059798320006002 PMC7271947

